# High-Resolution Ultrasound as a Key Tool for Dermatologic Surgery

**DOI:** 10.7759/cureus.101025

**Published:** 2026-01-07

**Authors:** Lucia Achell Nava, Juan Francisco Rodríguez Ramos, Claudia Gonzalez, Rodrigo Roldán, Gabriel Martínez Burillo

**Affiliations:** 1 Dermatology, Centro Medico Nacional 20 de Noviembre, Instituto de Seguridad y Servicios Sociales de los Trabajadores del Estado, Mexico City, MEX; 2 School of Medicine and Health Science, Instituto Tecnológico y de Estudios Superiores de Monterrey, Mexico City, MEX; 3 Radiology, Rosario University, Bogota, COL; 4 Dermatology, Clínica de Oncodermatología, Facultad de Medicina Universidad Nacional Autónoma de México, Mexico City, MEX; 5 Dermatology, Universidad Nacional Autónoma de México, Mexico City, MEX

**Keywords:** dermatology surgery, dermatology trends, high-frequency ultrasound, high-resolution ultrasound, skin cancer

## Abstract

Recent advancements in dermatologic imaging, such as dermoscopy and confocal microscopy, have transformed diagnostic and therapeutic approaches to skin lesions. In this context, high-resolution ultrasound (HRUS) has emerged as a valuable, accessible, and non-invasive modality for real-time visualization of cutaneous structures and skin pathology. Specifically, HRUS enables precise tumor margin delineation, supports biopsy guidance, and helps assess tumor extension, thereby optimizing surgical outcomes and reducing recurrence risk. While technical limitations and the need for operator expertise present challenges, continual technological advancements underscore its growing role in dermatology. This article reviews the applications of HRUS in enhancing surgical precision and outcomes through improved lesion characterization and procedural guidance. It provides a brief introduction to ultrasonography, its anatomical correlation, and representative clinical cases that illustrate its practical utility in dermatologic surgery. This narrative and illustrative review includes selectively chosen references emphasizing clinically relevant, high-impact studies, international consensus guidelines, and key illustrative publications. As high-frequency transducers and new ultrasound methods advance, clinicians will achieve even greater diagnostic and therapeutic precision. Nevertheless, dermatologists must continue to pursue specialized training in ultrasound-guided surgery and adhere to well-established protocols.

## Introduction and background

High-resolution ultrasound (HRUS) is a noninvasive, dynamic imaging modality that enables real-time visualization of the skin and its appendages. Its versatility and accessibility have increased its use as a key tool in dermatology over the past few decades. The ongoing development of new high-frequency transducers has enhanced resolution and the ability to analyze skin structures. Thus, alongside various modalities such as color Doppler and elastography, it has expanded its applications in diagnostics, pre-surgical planning, follow-up, and evaluation of various conditions. Sensitivity values of up to 97% have been reported for benign and malignant lesions, vascular anomalies, inflammatory and nail disorders, and cosmetic concerns. However, diagnostic performance depends on lesion type, ultrasound frequency, and operator expertise [1-6].

Background

Ultrasound has been used in dermatology for over 50 years, originally to measure skin thickness in dermatologic conditions and locate skin lesions [7,8]. Early applications were limited by low-frequency transducers and focused primarily on subcutaneous assessment, thereby restricting their clinical impact for many years. The subsequent development of high- and ultra-high-frequency probes over the past two decades enabled detailed visualization of epidermal, dermal, and appendageal structures, marking a turning point in dermatologic ultrasound. Recent technological advances and wider device access have greatly expanded its role, making ultrasound increasingly central to dermatologic surgery. Applications now range from preoperative assessment and planning to long-term follow-up [9]. It is also fundamental for other procedures, including ultrasound-guided biopsies, perioperative tumor delineation, and evaluating locoregional extension in malignancies.

Despite its advantages, ultrasound remains an operator-dependent technique, with diagnostic accuracy influenced by training, experience, and adherence to standardized protocols. As a result, global initiatives have focused on standardizing dermatologic ultrasound. Organizations such as Dermatologic Ultrasound (DERMUS) and the European Federation of Societies for Ultrasound in Medicine and Biology (EFSUMB) recommend a minimum frequency of 15 MHz for adequate skin evaluation [10,11]. HFUS (>20 MHz) and ultra-high-frequency systems (30-70 MHz) enable detailed visualization of structures, including sebaceous and apocrine glands [12]. The routine use of color Doppler is advocated for assessing inflammation and neovascularization [10,11].

Anatomical correlation

For accurate surgical planning of skin lesions, it is essential to distinguish normal skin anatomy. Generally, the epidermis typically appears as a hyperechoic band due to high keratin content, which strongly reflects ultrasound waves. In acral skin, a double band is seen because of the thick, keratin-rich stratum corneum. With age and photoaging, solar elastosis presents as a hypoechoic band in the upper dermis, resulting from elastin degradation and glycosaminoglycan deposition [13].

The dermis is also hyperechoic, though less so than the epidermis, due to collagen deposited by local fibroblasts. The hypodermis and subcutaneous cellular tissue appear hypoechoic due to the presence of fat lobules, allowing ultrasound waves to pass without resistance. Hair structures appear as oblique, bilayered, or trilayered hyperechoic images, while hair follicles are seen as hypoechoic structures that vary in depth depending on location and hair growth phase. For the nail apparatus, the nail plate is identified as a bilayered (dorsal and ventral) hyperechoic structure, the nail bed between the plate and the distal phalanx cortex as a hyperechoic line, and the nail matrix as a hypoechoic image surrounding the proximal area of the nail plate [13,14].

## Review

Application of ultrasound in dermatologic surgery

For surgical planning using ultrasound, it is important to identify key characteristics, such as whether the lesion is cutaneous or non-cutaneous in origin; its extension in longitudinal, transverse, and depth axes; whether it is solid or cystic; the presence of calcification or perilesional edema; vascularity; and involvement of deep planes. Some types of skin cancers, such as dermatofibrosarcoma protruberans, present characteristic ultrasound patterns that allow prebiopsy to suggest the type of tumor pathology [15]. This information aids the surgeon in assessing tumor infiltration, identifying lateral margins for a more appropriate surgical approach, and evaluating post-incisional margins, reducing recurrence risk and improving cosmetic outcomes. Additionally, ultrasound assists in monitoring recurrences and performing preoperative vascular mapping [16].

Non-melanoma skin cancer

Basal Cell Carcinoma

**Figure 1:**
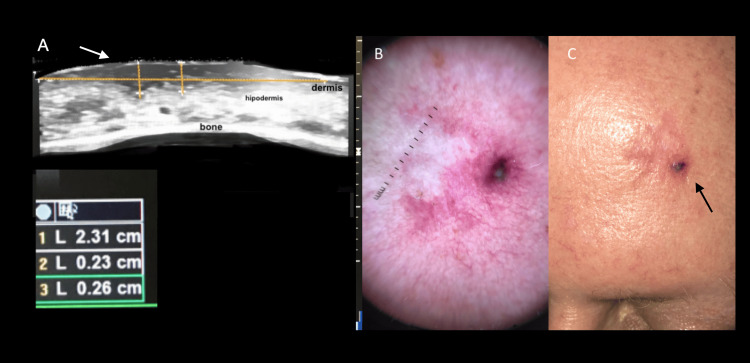
Ultrasound, dermoscopy, and clinical features of basal cell carcinoma. (A) High-resolution ultrasound shows a well-defined, hypoechoic, oval subepidermal lesion (white arrow). It involves the entire dermis and contains hyperechogenic punctiform areas. The horizontal yellow line shows the transverse diameter of the lesion; the vertical yellow lines show the tumor thickness. (B) Dermatoscopy shows a non-melanocytic lesion with arboriform telangiectasias. (C) Clinical image of the same patient (black arrow) shows the left frontal patch lesion with confirmatory biopsy of basal cell carcinoma.

In general, cutaneous neoplasms appear as hypoechoic lesions. Basal cell carcinoma (BCC) is the most common malignant skin tumor, comprising 75-80% of cases [17]. It grows slowly, has a low metastatic risk, but causes local destruction and invades nearby structures. There are four main histological subtypes, with the nodular subtype being the most frequent. Nodular BCC appears as a well-defined hypoechoic lesion in the dermis. In more aggressive subtypes, small hyperechoic dots, resembling “cotton flowers,” correlate with local microcalcifications (Figure 1).

**Figure 2:**
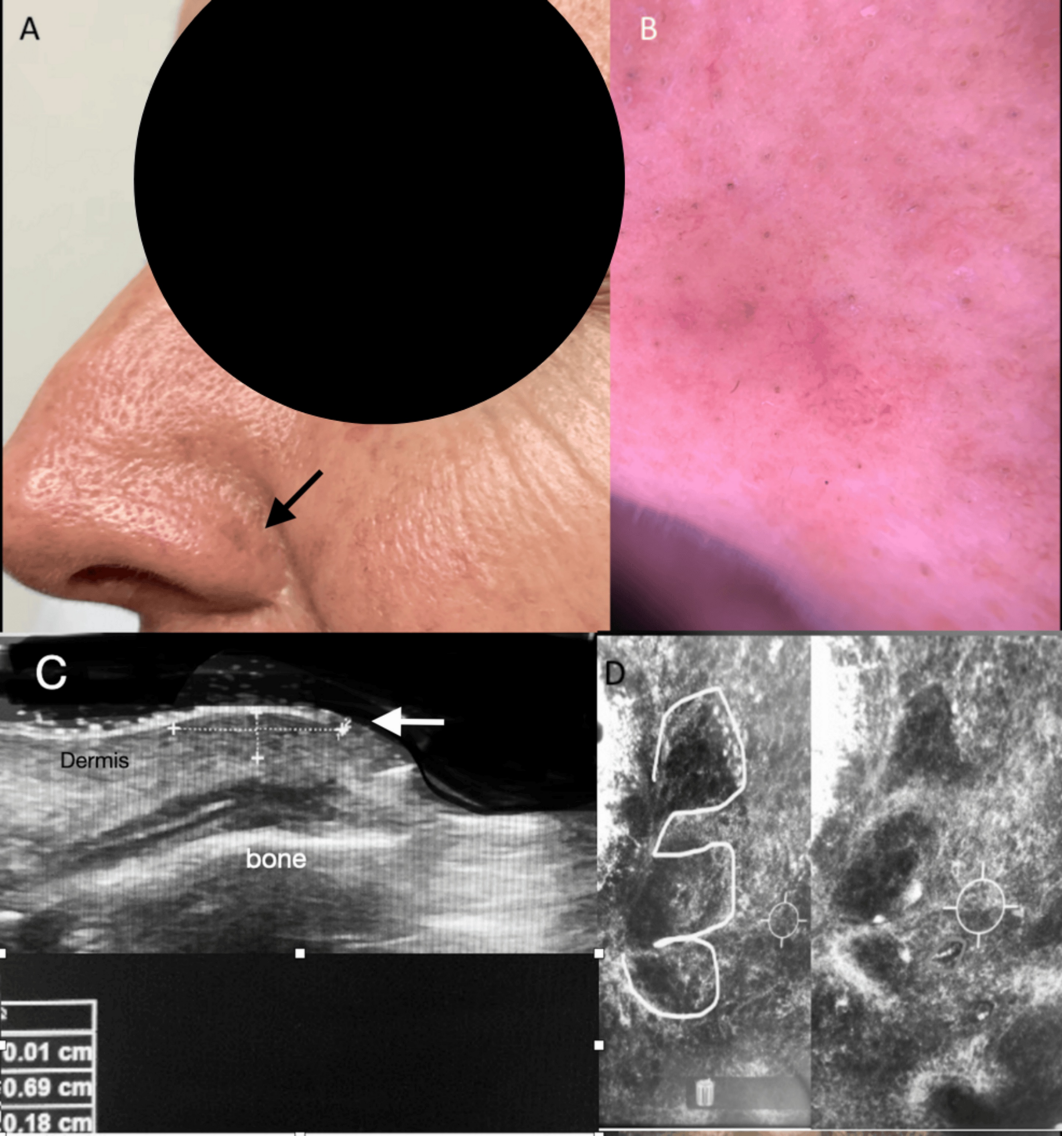
Multimodal assesment of basal cell carcinoma. (A) Skin-colored nodular lesion located on the nasal ala (black arrow), barely noticeable. (B) Dermatoscopy reveals a non-melanocytic, 5 mm, dome-shaped lesion with numerous small surface blood vessels (telangiectasias). (C) High-resolution ultrasound reveals a well-defined, subepidermal, hypoechoic lesion (white arrow) measuring 0.69 cm in width and 0.8 cm in depth, not invading the muscle plane or cartilage. (D) Confocal microscopy image displays nuclei elongated along the same axis (“nuclear polarization”), with tumor cell clusters showing a characteristic “peripheral palisade” pattern, and separation from the surrounding stroma. These findings confirm the diagnosis of basal cell carcinoma.

Ultrasound has shown a sensitivity of 96% and specificity of 84% for preoperative BCC assessment, enabling a more conservative approach and ruling out deeper tissue invasion, which may alter the therapeutic strategy [18,19] (Figure 2).

Squamous Cell Carcinoma

**Figure 3:**
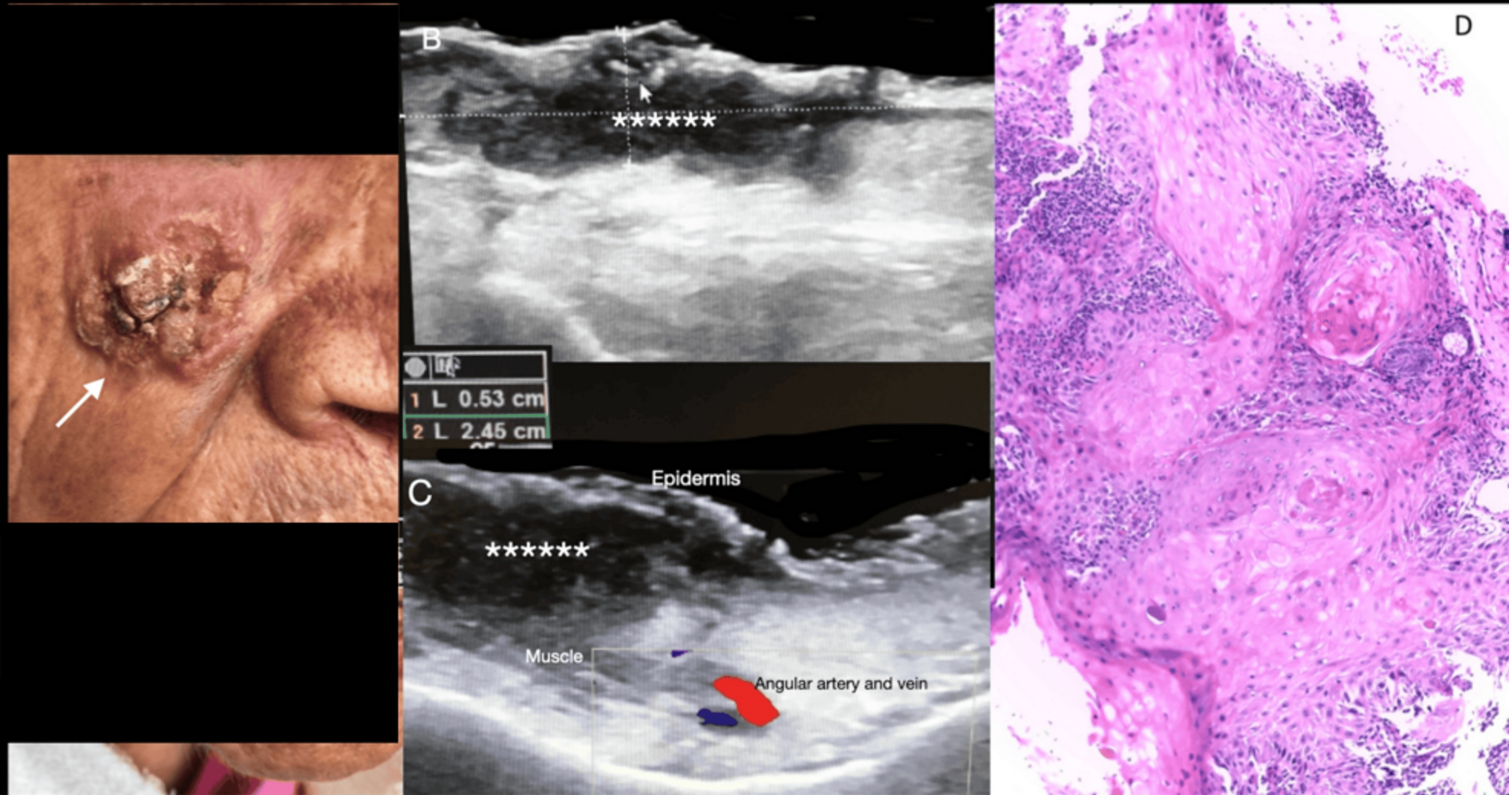
Clinical, ultrasound, and histopathological features of invasive squamous cell carcinoma. (A) Invasive squamous cell carcinoma (white arrow) in a 97-year-old female. (B, C) High-resolution ultrasound depicts an irregular, hypoechoic mass (marked by white asterisks) that invades the dermis, subcutaneous tissue, and muscle, resulting in loss of the usual skin structure. (B) White lines indicate the width and depth of the mass. (D) Histopathological image shows loss of the outer stratum corneum, disruption of normal dermal architecture, dyskeratotic keratinocytes, atypical mitoses, acantholysis, and keratin pearl formation.

Squamous cell carcinoma (SCC) also appears as a homogeneous hypoechoic lesion with prominent central and peripheral vascular flow. Preoperative evaluation is particularly useful for identifying high-risk SCC characteristics, such as invasion beyond 6 mm, infiltration, vascular pattern, posterior enhancement, and other features that may predict metastatic risk or recurrence in follow-up cases (Figure 3).

**Figure 4:**
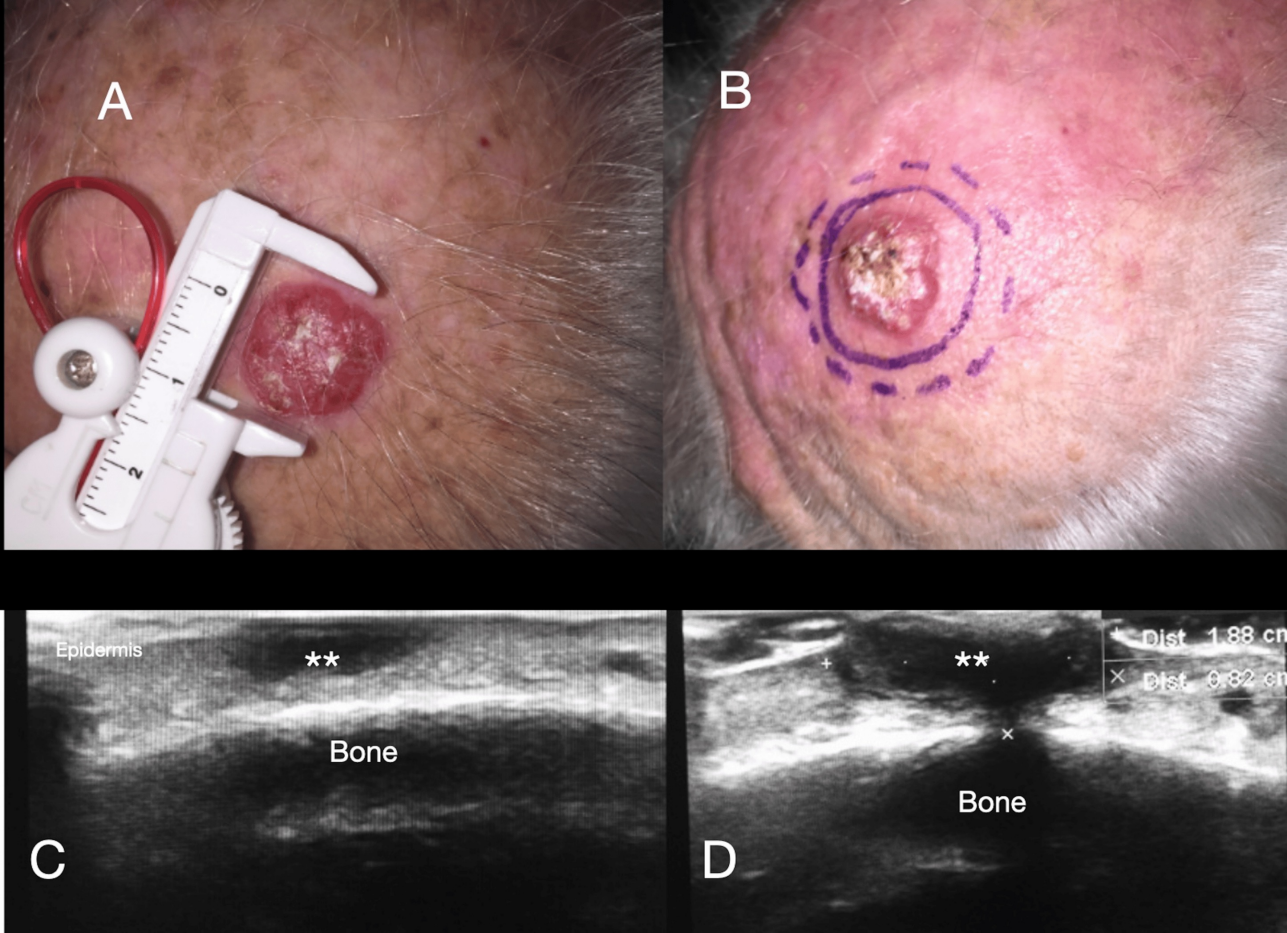
Sonographic evaluation of a nodular squamous cell carcinoma. (A, B) An 82-year-old male with a 1.6 cm nodular lesion on the scalp. The lesion is skin-colored and has a keratinized surface. The histopathologic report confirms a well-differentiated squamous cell carcinoma. (C, D) High-resolution ultrasound images in axial and longitudinal views show the lesion. The white asterisks (***) indicate a well-defined hypoechoic nodular lesion extending to the deep plane up to the fascia. The posterior acoustic shadow is an artifact caused by superficial keratin. In image D, calipers (+) measure its longitudinal diameter, and calipers (x) indicate the tumor thickness.

However, the diagnostic utility of ultrasound is limited in hyperkeratotic lesions due to the stratum corneum’s keratin, which interferes with ultrasound waves; therefore, removing the keratin component is recommended to improve ultrasound visualization [19] (Figure 4).

Melanoma

**Figure 5:**
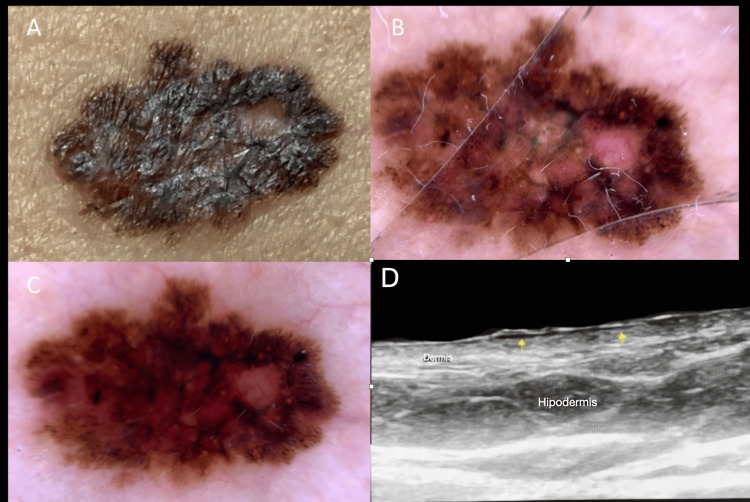
Dermoscopic and ultrasound features of a vertically growing melanoma. (A) Clinical examination shows a patchy, pigmented lesion. (B, C) Dermoscopy reveals a poorly defined, melanocytic lesion with an irregular pigment network, white areas, and a small, unpigmented, raised area with telangiectasias on its surface, suggesting vertical growth. (D) High-resolution ultrasound images show yellow arrows pointing to a well-defined, hypoechoic area in the upper dermis, which elevates the surface skin and indicates vertical growth. This ultrasound guided the biopsy, which confirmed superficial spreading melanoma.

Melanoma may appear as a fusiform or oval hypoechoic lesion. It should be noted that melanoma does not have specific patterns and may be limited, as melanin is not distinguishable by ultrasound. The most important prognostic factor is vertical growth, assessed by the histological Breslow index and dermal invasion. Even in melanomas with superficial extension, we can correlate the depth and extension, which is valuable for staging and surgical planning (Figure 5).

Studies report higher sensitivity and specificity with high-resolution ultrasounds (>20 MHz). This assessment enables not only the determination of prognosis but also the establishment of safe surgical margins and the identification of indications for sentinel lymph node biopsy [19,20].

Benign lesions and nail apparatus

Benign Lesions

For benign lesions, ultrasound is also applicable. Seromas, which commonly form after extensive excisions or biopsies, appear as anechoic, compressible, and avascular lesions with serous content. Ultrasound supports planning for aspiration or surgical drainage. Hematomas appear as anechoic collections with peripheral vascularization, though echogenicity varies with time, making clinical history essential. Lipomas are well-defined, oval, hypovascular lesions with hyperechoic septa, while epidermoid cysts appear as well-circumscribed, round-to-oval nodules with a hypoechoic tract connecting to the epidermis, posterior enhancement, perilesional inflammation, and hypoechoic fluid around the lesion, which is valuable for pre-surgical assessment and planning [21,22].

Nail Apparatus

**Figure 6:**
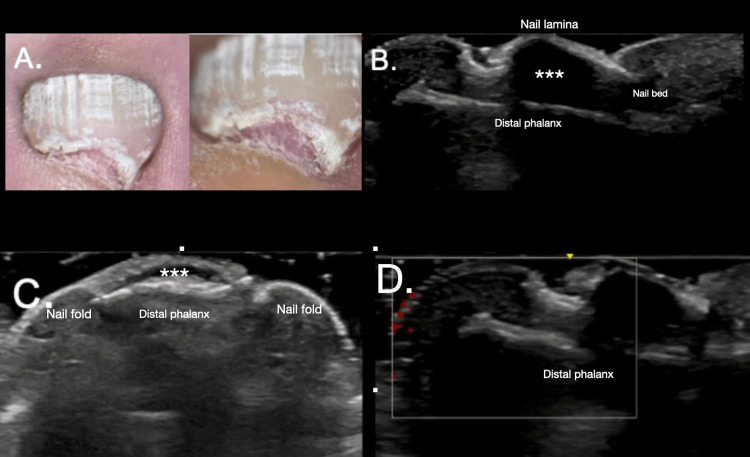
Imaging features of a subungual lesion. (A) Clinical image and LUMIO augmentation show the nail apparatus of the left first toe with a firm, pink lesion located below the nail plate, displacing it upwards into the nail lamina. The patient underwent high-resolution ultrasound evaluation to rule out a glomus tumor. (B-D) High-resolution images of the affected nail’s ungual apparatus reveal a wavy lamina displaced upwards, with a hypoechoic nodule (marked by white asterisks **) occupying the nail bed. The lesion is expansive, extends to the distal phalanx without affecting it, and appears avascular on Doppler evaluation, consistent with a fibrous nail bed. In image C, the nail folds are thickened, indicating chronic inflammation secondary to recurrent onychocryptosis.

For common benign entities of the nail apparatus, ultrasound imaging is often required. Traditionally, MRI has been the preferred study for nail lesions; however, MRI is less accessible, more expensive, and provides only delayed results, delaying diagnosis [23]. Currently, most tumor and non-tumor lesions of the ungular apparatus have characteristic ultrasound patterns that allow for the precise establishment of the etiopathological diagnosis of the entity and perform an adequate approach and surgical planning. Ultrasound can evaluate subungual tumors and their relationship with the nail matrix, aiding in surgical planning and enabling detection of lesions as small as <2 mm, which is particularly helpful in the differential diagnosis of subungual tumors such as glomus tumors. On HRUS, glomus tumors, originating from the neuromyoarterial receptors of the nail bed, typically appear as small, well-defined hypoechoic lesions with marked hypervascularity on Doppler imaging and occasional distal phalanx erosion, whereas benign inflammatory conditions such as recurrent onychocryptosis usually demonstrate avascular or hypovascular hypoechoic changes of the nail bed, nail plate distortion, and periungual soft-tissue thickening without bone involvement [24] (Figure 6).

## Conclusions

Ultrasound is a versatile and accessible tool that has expanded its application in modern dermatology. However, it is important to know that there is an operator dependence. Its ability to provide real-time imaging, guide invasive procedures, plan surgical interventions, and facilitate follow-up has established its value in the management of a wide range of cutaneous pathologies. The continuous development of high-frequency transducers and the integration of new ultrasound modalities hold promise for improving diagnostic and therapeutic precision. In parallel, emerging non-invasive imaging techniques such as optical coherence tomography and photoacoustic imaging are gaining interest by enabling near-histological structural assessment and functional tissue characterization, potentially complementing HRUS. Nonetheless, it is crucial to promote specialized, ongoing, and up-to-date training for dermatology specialists who use ultrasound in dermatologic surgery, based on protocols that have been described and widely validated since 2016. In summary, HRUS bridges histopathology and imaging, making it an essential tool for surgical planning and the management of dermatologic diseases.
